# Science Incubators: Synthesis Centers and Their Role in the Research Ecosystem

**DOI:** 10.1371/journal.pbio.1001468

**Published:** 2013-01-15

**Authors:** Allen Rodrigo, Susan Alberts, Karen Cranston, Joel Kingsolver, Hilmar Lapp, Craig McClain, Robin Smith, Todd Vision, Jory Weintraub, Brian Wiegmann

**Affiliations:** The National Evolutionary Synthesis Center, Durham, North Carolina, United States of America

## Abstract

How should funding agencies enable researchers to explore high-risk but potentially high-reward science? One model that appears to work is the NSF-funded synthesis center, an incubator for community-led, innovative science.

The Biological Sciences Directorate is the only directorate at the National Science Foundation that presently funds synthesis centers. As originally conceived, synthesis centers were meant to focus on the re-use of existing data to address “big picture” questions [1]. The National Center for Ecological Analysis and Synthesis (NCEAS) was the first synthesis center to be established in 1995. Since then, three more synthesis centers have been funded: our Center (the National Evolutionary Synthesis Center, or NESCent), the National Institute for Mathematical and Biological Synthesis (NIMBioS), and most recently, the Socio-Environmental Synthesis Center (SESYNC). The iPlant Collaborative (iPlant) also shares some aspects of the synthesis center model.

Under current policy, the National Science Foundation (NSF) will fund synthesis centers for a maximum of 10 years. One argument for this limit is that the Foundation funds innovation; long-term funding of any project, whether it is one investigator's research or a large center, is anathema to this principle (interestingly, there does not appear to be any obvious limit to the number of times institutes—as opposed to centers—can compete for renewed funding). This is a powerful argument, particularly since the rationale for this line of reasoning that is often advanced is that long-term support removes funds from the pool available to support new projects. A reasonable counterargument—and one that we make in this article—is that synthesis centers are actually places where innovation happens, in ways that avoid the multiplication of resources and infrastructure that are funded through capital and indirect costs on individual grants. Therefore, long-term and sustained support for synthesis centers falls quite nicely within the mandate of the NSF to support novel research in cost-effective ways.

Synthesis centers do not support the collection of new data; instead, they add value to the data already collected across a diverse and extensive suite of research projects spanning a range of disciplinary and subdisciplinary domains. But the solutions to “big picture” problems do not come cheap—they typically require the recruitment of new ideas and practitioners from disciplines outside the primary area of focus. At synthesis centers, interactions between data scientists—statisticians, mathematicians, computer scientists, and computational biologists—and scientists with domain-specific knowledge are central. But how does one facilitate collaborations that require researchers to work through the nuances of discipline-specific vocabularies, assumptions, concepts, and methodologies? If there is one truth that synthesis centers have learnt, it is that this cannot be done by e-mail.

As the first synthesis center to be established, NCEAS developed a model for continually nurturing new collaborations that have an impact above and beyond what could be achieved by individual investigator grants [2],[3]. The role of synthesis centers has continued to evolve as more centers have been established, but in our opinion, the following four principles of the synthesis center model are still fundamental: (1) cross-disciplinary, synthetic science requires real-time and face-to-face interactions; (2) the big questions in any given discipline should be identified by its community of practitioners; (3) the big questions in any discipline change, as do the skills that are needed to address them, and we need to respond flexibly to these changes; and (4) if the science itself is high risk, but with potentially high rewards, then we need to make sure that the risk of failure is not amplified by a lack of logistic and informatics resources, and/or administrative distractions. Together, these principles establish a coherent argument for the value of synthesis centers and their role in the research ecosystem. Together, they also broadly define the goals of synthesis centers as these apply to data, methods, ideas, disciplines, and people: to connect, share, and transform.

The article by Sidlauskas et al. [4] discusses what synthesis is—essentially, vertical integration across different types of data within a discipline and/or horizontal integration across two or more disciplines where the objects of synthesis are methods, concepts, and knowledge. At NESCent, our focus is on evolutionary science and, in particular, the synthesis within and across disciplines that span the natural, medical, and social sciences, to deliver new and potentially transformative ways of understanding the unity and diversity of life, writ large. The scientific community itself decides what is new, what is exciting, what is innovative in evolutionary science. Multiple times a year, we invite researchers to submit proposals for working groups, meetings, short-term research, and postdoctoral and graduate student projects, and these are reviewed by members of our Advisory Board, who themselves are drawn from the wider evolutionary science community. NESCent does have thematic programs, whereby proposals in certain domains are solicited, but even these themed initiatives come from the community, via our Advisory Board, community summit meetings, or informal communications from evolutionary scientists. In this way, the science at the center is guided by the scientists themselves, scientists who understand how their discipline is changing, what the emergent questions are, and what skills may be required to deliver solutions. By virtue of this open and responsive process, NESCent is justifiably seen as a community resource.

Although NESCent supports the scientific community in a variety of ways, it is not a funding agency. The activities and people we support align with our mission and with community needs. Our Advisory Board evaluates proposals for meetings, postdoctoral, graduate, and sabbatical fellowships, not just on the transformative quality of the science, but also the demographic, disciplinary, and career-stage diversity of those who apply, and the extent to which the participants come from countries outside the United States. The projects and activities we support represent collaborations between the scientists and NESCent.

NESCent and other synthesis centers are also places for community building. The ever-changing community of postdoctoral fellows, sabbatical scholars, short-term visitors, and graduate students produces a uniquely stimulating environment for knowledge exchange and the generation of new ideas. A brick-and-mortar center in which the physical and disciplinary juxtaposition of scientists connects them to a range of research activities serves to break down the conceptual, linguistic, and cultural barriers that separate disciplines. Although we may speak of evolutionary science as if it was a single, cohesive discipline, with well-formulated ways of working and of understanding the changing world, biologists know that this is not true. There is a great deal of diversity, manifest not only in the types of questions that evolutionary scientists ask but also in their requirements with respect to the data they collect, analyze, and store. We study the histories of genes, pathways, genomes, metagenomes, organisms, species, communities, ecosystems, languages, religions, government, and cultures. The highly interactive environment at NESCent makes it difficult for, say, a marine biologist to avoid speaking to paleontologists [5] and evolutionary ecologists to remain unaware of the research of those working on computational phylogenetics [6]. The ideas that emerge from this creative mixing are not entirely predictable, and indeed that is part of the point.

Furthermore, by bringing together researchers who have been sufficiently motivated to submit proposals for meetings and working groups, NESCent and other synthesis centers also foster the development of self-identified collaborative communities, comprising groups of individuals working on similar problems but with complementary data, methods, and practices. There are many reasons why fostering the development of these “communities of practice” [7],[8] is important, but here are two of the most compelling.

First, communities of practice are needed to help guide the development of software, databases, and other cyberinfrastructure, including the human capacity to effectively exploit and develop cyberinfrastructure. To promote this at NESCent, both our resident community and the scientists who visit for meetings collaborate closely with specialist informatics staff at the center to identify the informatics needs for their research and help assemble the tools required to reach their scientific goals. By embedding informatics specialists within the community of practice, and cultivating expertise within that community, we ensure that any new software and database resources that get developed, and any modifications that are made to existing ones, are scientifically useful. The Center's informatics staff, which has experience meeting the needs of many different projects, is motivated to ensure that software and database development is not duplicative, to connect researchers with similar needs, and to develop solutions that merit continued maintenance and development by the community long after the center's investment has concluded. Both the Center and the individual investigators who are organized into communities of practice make complementary contributions to this process, which would be far less effective with either the Center or with the individual investigators acting in isolation from each other.

Second, by evaluating proposals not just on the quality of the science proposed, but also on the career-stage and demographic diversity of participants, the center is in a position to encourage nascent communities of practice to engage both seasoned and emerging investigators as well as those investigators who may encounter cultural, financial, and institutional challenges that diminish the opportunities for scientific research and/or interdisciplinary collaboration. Participation by these groups has two important downstream effects: (1) these communities of practice end up with a self-sustaining pool of energetic scientists who are able to disseminate and promote the new ideas that emerge to their students and the next generation, and (2) because synthetic science (as practiced at synthesis centers) is relatively inexpensive, scientists who are part of these communities can still do exciting research, even if they work at institutions where time and money cannot easily be found to support wet labs. In this way, these communities of practice have the best opportunity to reach across the generations of scientists and increase awareness of their science in demographic communities of investigators that would otherwise be hesitant to participate or ignorant of what is on offer.

So, as a synthesis center, NESCent asks the community to identify exciting, high-risk/high-reward synthetic research directions; brings a diversity of researchers to the center so they have the opportunity to iron out any cross-disciplinary wrinkles face-to-face; and where appropriate, engage researchers with informatics professionals to identify and develop their own informatics resources. And as an added bonus, all the operational and logistic arrangements—travel, accommodation, meal arrangements, meeting planning, and facilitation—are made by the center staff, so that researchers only have to think about the science. In short, NESCent and other synthesis centers catalyze collaboration and make it easy for people to work together to get really interesting things done.

This model for a synthesis center is strikingly similar to a business “incubator,” which provides critical legal, administrative, and financial services to start-up enterprises so that they may move from conception to commercial viability [9],[10]. Similarly, synthesis centers provide opportunities for scientific teams to explore ideas that are risky, that require novel combinations of expertise, or that need some informatics investment to be realized. Just as business incubators improve the chances of start-up companies to secure investment, synthesis centers invest in early stage ideas, databases, papers, and other products that researchers need to demonstrate proofs-of-concept (and secure further funding) to naturally cautious grant reviewers and funding agencies.

NESCent has been quite successful scientifically: In the eight years of our operation, our participants have produced more than 500 publications, garnered more than 8,000 citations, and we have a Center *H*-index of 43 (a more detailed analysis of our performance can be found in NESCent's Assessment Report [11]). And it has also been successful as a science incubator. Recently, we asked scientists who participated in NESCent activities or who have been resident at NESCent about the amount of funds they had received from grants as a direct consequence of their involvement in NESCent. The total amount was more than $24 million, almost matching the amount of funds that NESCent received from the NSF for its core activities, a 1-to-1 return on investment. Note that this total does not include the close to $10 million of funds awarded to NESCent staff for projects mostly related to educational outreach and the development of community informatics resources.

Everyone wishes for success, of course, but an equally important role for incubators is that they allow for quick and low-cost failure. It is useful to know when a project cannot succeed despite having the best opportunities to do so. A synthesis center is a cost-effective vehicle for vetting high-risk, high-impact science. Through the Center's investment, less is personally at stake for the researchers, so that they are more likely to take intellectual risks and can easily move on to the next idea should the first one not pan out.

There have been several commentaries on the need to think about smart ways to provide funds and opportunities to explore innovative but risky ideas. James Langer, a former Vice President of the U.S. National Academy of Sciences, wrote recently that “the funding agencies, especially NSF and the DOE, must admit that it is not humanly possible to predict, with high accuracy, which research projects ultimately will have the most impact. Peer review, at best, can identify fundable proposals. When there are too many of these, as at present, agencies must find other ways to decide which to support. … At the very least, it will be critical to find modes of operation that do not discourage the most imaginative investigators just because their proposals are too innovative” [12]. It seems to us that synthesis centers—and indeed, other centers that function as science incubators—are the perfect vehicles for achieving these goals. Given the importance of synthesis centers in establishing communities of practice, facilitating bottom-up developments in cyberinfrastructure, and providing a cost-effective means of vetting high-risk/high-impact science, one could make a case that it would be in the best interests of the NSF to allow for the option of funding such centers indefinitely (subject, of course, to periodic review and competitive renewal). As we noted above, although the generic argument against long-term support rests on the threat of a diminishing pool of funds available for new and innovative research, it is difficult to understand why there is a distinction at the NSF between the strict funding lifespan of “centers” and the apparently unrestricted funding opportunities available to “institutes.” Indeed, whether we call something a center or an institute or an infrastructural resource obfuscates the key point of this article. To support synthesis centers is to support innovative research, whereas to lose a successful synthesis center is to lose the institutional knowledge—the “incubating” know-how, as it were—to build communities by connecting people, sharing ideas and data, and ultimately transforming science.

## Abbreviations

iPlant: iPlant Collaborative
NCEAS: National Center for Ecological Analysis and Synthesis
NESCent: National Evolutionary Synthesis Center
NIMBioS: National Institute for Mathematical and Biological Synthesis
NSF: National Science Foundation
SESYNC: Socio-Environmental Synthesis Center
